# A qualitative phenomenological analysis of the subjective experience and understanding of the at risk mental state

**DOI:** 10.1080/17482631.2017.1342504

**Published:** 2017-07-10

**Authors:** Benjamin Brew, Ciaran Shannon, Lesley Storey, Adrian Boyd, Ciaran Mulholland

**Affiliations:** ^a^ School of Psychology, Queens University Belfast, Belfast, Northern Ireland; ^b^ STEP Team, Northern Health and Social Care Trust, Antrim, Northern Ireland; ^c^ School of Medicine, Dentistry and Biomedical Sciences, Queens University Belfast, Northern Ireland

**Keywords:** Qualitative analysis, interpretative phenomenological analysis, subjective accounts, psychosis, ultra-high risk, prodrome, At Risk Mental State

## Abstract

Over recent years there has been a growing interest in identifying the early stages of psychosis. The At Risk Mental State (ARMS) is characteristic of the prodromal stages of psychosis and its identification gives rise to a number of clinical and research opportunities including early intervention and prevention of psychosis. This study employs interpretative phenomenological analysis to gain insights into the subjective experience and individuals understanding of the development of their ARMS. Five participants took part and provided information on the experience of symptoms, life prior to onset of their ARMS and their understanding of symptoms and their development through a semi structured interview. From the analysis of transcripts eight themes emerged which were common across participants accounts. Three themes of experience (disturbed world/disturbed self, disconnection with the world, thunderstruck) and five themes of understanding (absence of understanding, use of others, identity, forming links, fragmented understanding) were identified. Themes are explored in detail and discussed in relation to existing literature and theory. Clinical implications, directions for future research, and limitations are discussed within.

## Introduction

In recent years there has been increased interest in the early stages of psychosis in both research and clinical practice. Developments in the field have allowed for the identification of, and early intervention with, individuals who are presenting with an increased risk of developing a psychotic illness. Yung and colleagues (2003, 2004) coined the term At Risk Mental State (ARMS) to refer to the phase that was characteristic of prodromal psychosis to assist in the identification of such individuals. The Personal Assessment and Crisis Evaluation (PACE) clinic pioneered much of the early work in ARMS research and developed measurement tools, and accompanying criteria to identify young people who were ultra-high risk (UHR) of developing psychotic illness (O’Connor, 2013). UHR criteria requires a person to be aged between 14 and 30 years and meet one or more of the following groups: (1) experienced attenuated psychotic symptoms during the past year; (2) experienced brief limited intermittent psychotic symptoms that have lasted no longer than 1 week and have subsequently lessened or ceased; (3) have a first degree relative with a psychotic disorder, or have been identified as having a schizotypal personality disorder and have experienced a decline in functioning over the past year. Early research using these criteria reported rates of transition to first episode psychosis (FEP) ranging from 35% to 54% over a 1 year period (Nelson, Yung, Bechdolf, & McGorry, 2008), providing substantial support for the validity of the criteria. However, over more recent years a decline in transition rates to FEP has been observed (for a discussion of this, see Hartmann et al., 2016).

The use of UHR criteria gives rise to a number of clinical and research opportunities, including early intervention and detection of psychotic illness. Given that the ARMS is a relatively new concept, it is important to gain deeper insight into the subjective experience of symptoms to develop clinical understandings of them. In a review of UHR research, Nelson and colleagues (2008) argued that a key step in approaching any psychopathological state is to recreate its experiential dimensions, to enable clinicians to gain an understanding of phenomena they are dealing with. However, to date relatively little research has focused on the subjective experience of the ARMS. What research that has been conducted has reported isolation, difficulty with social interaction, and a tendency to keep experience of symptoms to themselves (Byrne & Morrison, 2010; Welsh & Tiffin, 2011). Although there is a dearth of research on the subjective experience of the ARMS, we can look towards the literature in FEP for further insights, where individuals have met criteria for a full psychotic episode as opposed to being identified as being at risk for one. In a review of qualitative studies in FEP, Boydell, Stasiulis, Volpe, and Gladstone (2010) reported retrospective accounts of the prodromal phase of illness were characterized by a wide range of symptoms and behaviours, including sleep disturbances, attentional difficulties, social withdrawal, and a loss of self which often resulted in job loss or academic failure. Further research has highlighted that psychosis is much more than its symptoms describing the losses in self, consensual reality, security and relationships, and the pain that these cause (McCarthy-Jones, Marriott, Knowles, Rowse, & Thompson, 2013). In their qualitative study of psychotic experiences in young men following FEP, Hirschfeld, Smith, Trower, and Griffin (2005) reported three main categories of psychotic experience; the occurrence of uniquely psychotic phenomena, experiencing emotions, and thinking about dying. These themes were interwoven and described participants altered interactions with the world around them in response to their psychotic symptoms, and highlighted the emotional impact and personal and interpersonal changes as a consequence of psychotic symptoms.

With regards to developing an understanding, within the literature there is no conclusive theory to explain the development of psychosis, however a number of theories and models have been proposed. Garety, Kuipers, Fowler, Freeman, and Bebbington (2001) proposed a cognitive model for the positive symptoms of psychosis. Their model posits a central role for emotion, where disturbed affect leads to maladaptive psychotic appraisals of unusual experiences or life events which become reinforcing of themselves, and in turn maintain further psychotic appraisals. Bio-psychosocial models of psychosis have also been proposed, with the diathesis-stress model being one of the main models of understanding. It proposes environmental stressors interact with underlying biological vulnerabilities, which in turn leads to the development of psychosis or psychotic like experiences (Walker, Mittal, & Tessner, 2008). However the biological aspect of this model has been suggested to be reductionist with the prominence of late adolescent onset being neglected. The period of adolescence and young adulthood which UHR criteria include is a transitory period, which is significant for identity development and formation (Erikson, 1968). It is characterized by individuals becoming less dependent on family, and more dependent on peers for development and support (Mackrell & Lavender, 2004). Harrop and Trower (2001) proposed a theory of blocked adolescence which argues psychosis which emerges in late adolescence is a disorder of adolescent development, where individuals struggle to meet the basic needs of adolescence, namely developing autonomy and individuation from family, and forming peer relationships. They suggest psychosis may be a consequence of these thwarted needs, where individuals are unable to fully separate from their parents, viewing them in an idealized fashion and are unable to form attachment to peers.

An important aspect of gaining a deeper insight into the subjective experience of the ARMS is understanding how people make sense of their symptoms, and this is an important factor for services to consider when engaging with a person. Research in the subjective understanding of mental illness has identified numerous factors which individual’s draw on, including physical and biological, social and environmental, and personal and individual factors (Baker & Procter, 2013; Elliott, Maitoza, & Schwinger, 2011; Williams & Healy, 2001). It is well documented that beliefs about health and illness shape our emotional and behavioural responses to illness and our relationships with healthcare providers (Salmon, 2002). Research suggests when individuals receiving care share the same explanatory model of illness as the clinicians working with them, it strengthens the therapeutic relationship and increases service user satisfaction (McCabe & Priebe, 2004). A variety of theoretical models have been proposed to link health beliefs to a range of different health behaviours, such as self-regulation theory (Leventhal, Nerenz, & Steele, 1984) and the health belief model (Becker & Maimon, 1983). However these have developed in the context of physical health and researchers in mental health argue they do not directly apply to mental health difficulties. These models are formed upon the idea that health beliefs are sufficiently stable over time for predictions to be made on their basis, which is not necessarily the case with mental health.

Williams and Healy (2001) examined the perceptions of illness causation of people who had been referred to a community mental health team. Their results indicated a sense making process where individuals drew on a range of co-occurring, and at times contradicting understandings, suggesting that the process of finding meaning is characterized by movement and uncertainty as individuals hold a variety of explanations simultaneously, and move rapidly between them. From their findings they suggest an exploratory map of understanding mental illness rather than an explanatory model. In support of this, in their review of qualitative studies in FEP, Boydell et al. (2010) reported that young people engage in a search for meaning for their symptoms, adopting multiple explanations which change over time. Judge, Estroff, Perkins, and Penn (2008) reported individuals recovering from FEP described not initially seeking help, understanding psychotic symptoms as normal at first and simply part of who they were, reflecting an underlying confusion between psychotic symptoms and their sense of self. Participants in their research then went on to develop explanatory models, drawing on psychosocial events, cultural factors and personal experiences. Further research in individuals understanding of psychosis draws on a range of factors including drug use, adult and childhood trauma experiences, personal characteristics (Dudley, Siitarinen, James, & Dodgson, 2009) and difficulties adjusting to, and negotiating age related milestones and developing relationships, independence and personal responsibility (Hirschfeld et al., 2005). Furthermore, explanations used to help form understandings of psychosis are often linked up in an individual’s sense of themselves prior to the onset of illness (Hirschfeld et al., 2005).

Given the lack of theory about the ARMS and transition to FEP, it is important to develop a deeper understanding of this clinical phenomenon to inform theory development and highlight areas of consideration. To date little research has focused on the subjective experience and understanding of the ARMS, and most research in the area has been conducted retrospectively with individuals recovering from FEP. This study aims to address this gap in literature by employing interpretative phenomenological analysis (IPA) to the research questions “How do individuals experience their ARMS?” and “How do individuals make sense of the development of their ARMS?”

## Methods

### Ethical approval

Participants were initially approached by members of their clinical team who provided them with a brief summary of the study and an accompanying information sheet. Participants were given a 2 week period to register an interest in the study by returning a consent to be contacted form in a provided stamped addressed envelope. Prior to the commencement of interviews, participants were provided with a further explanation of the study, informed of their rights as a participant and given an opportunity to ask questions. Participants’ capacity to consent was assessed by clarifying their understanding of the research and what their participation involved before asking them to sign an informed consent form. Participants were informed they had the right to withdraw from the study at any time throughout the interview, and were given a further 48 h period following the interview to withdraw from the study, with any decisions they made about their participation having no impact to the care offered to them, both at present and in the future. After the completion of the research interview, participants completed a debriefing procedure, and were given further opportunity to ask questions about the study and their participation.

Ethical approval was granted on 23rd April 2015 by the Office for Research Ethics Committees Northern Ireland (Reference code 15/NI/0034), which is the regional research ethics committee regulated by a statutory research governance framework.

### Participants

Participants were recruited from the Service Treatment and Education and Prevention (STEP) Team in the NHSCT in Northern Ireland, which provides assessment and intervention for individuals who meet UHR criteria. Inclusion criteria for this study required participants met criteria for an ARMS as assessed by the Comprehensive Assessment of At-Risk Mental State (CAARMS; Yung et al., 2005) and were in the attenuated symptoms group of UHR criteria, were aged between 16 and 24 years and were under the care of the STEP team. Participants were excluded from the study if they were unable to provide informed consent due to actively experiencing a disturbance in mental state, or if their understanding of the English language was insufficient to provide informed consent or engage in the research interview. Utilizing these criteria ensured a homogenous sample which was representative of an ARMS population and appropriate for IPA.

Six people registered an interest in the study, but one withdrew interest at a later date. In total five people took part, one female and four males. Ages ranged from 17 to 20 years. All participants had been engaged with the STEP team for less than 1 year, were under regular psychiatric review, and were offered or engaged in psychological therapy. This provided a homogenous sample suitable for IPA. The sample size is in line with Smith and Osborn’s (2003) recommendations of five or six participants for an IPA study, allowing for the in-depth analysis of data whilst ensuring data generated is of a manageable size. For further descriptive information regarding participants see Table I. All participants’ names have been changed to ensure their anonymity.

**Table I.** Participant information.

Participant	Sex	Age in years	Employment status
Beth	Female	20	Student
Paul	Male	20	Student
Steven	Male	20	Employed*
Trevor	Male	17	Student
Joseph	Male	19	Employed*

* Denotes currently on sick leave.

### Materials

All participants had completed the CAARMS (Yung et al., 2005) prior to being accepted into the care of the STEP team. As such each participant in this study had recently been assessed using the CAARMS by clinicians working within the team. The CAARMS is a semi-structured interview used to assess UHR status and to measure a range of sub-threshold symptoms associated with the prodromal phase of psychosis. It is grouped into seven scales: positive symptoms; cognitive change; emotional disturbance; negative symptoms; behavioural change; motor/physical changes; and general psychopathology, with scores for each ranging from 0 to 6 for both frequency and intensity of symptoms. The psychometric properties of the CAARMS have been well established with Cronbach’s alpha values of 0.85 being reported, indicating it has a high level of internal coherence (Raballo, Nelson, Thompson, & Yung, 2011). Yung and colleagues (2005) have also demonstrated the validity and reliability of the measure, demonstrating both high levels of interrater reliability and the predictive validity of the CAARMS.

Participants were given a participant information sheet prior to registering an interest in this study. Semi structured interviews were conducted using an interview schedule with relevant prompts to guide the interview process. Questions focused on the experience of symptoms, life prior to onset of their ARMS and individuals understanding of symptoms. Questions were developed to encourage participants to give an account of their symptoms and life experiences, and their understanding of them. These were considered to have a phenomenological status (Charmaz, 1990) in that they represented individuals’ perspective of their experience rather than an objective truth. Interviews were recorder on a digital audio recorder and later transcribe using transcription computer software and a foot pedal.

### Procedure

Interviews were held in a clinical area which participants were familiar with and lasted between 50 and 110 min in length.

### Data analysis

Following the transcription of interviews, each transcript was analysed guided by the methods of IPA outlined by Smith, Flowers, and Larkin (2009). Firstly this involved reading and re-reading transcripts to gain familiarity with each. Initial notes were recorded by the researcher, which were then discussed with the research team to check for any bias that was emerging in the initial analysis. Transcripts were then approached again and more detailed notes on the overall transcript, and segments of it, were made including comments on recurring patterns and associations, use of language, the emotional content and the researcher’s reaction to the text. The researcher then used these alongside transcriptions to document emerging themes in the data whilst remaining cognisant of the double hermeneutic process of analysis and endeavouring to keep themes grounded in data. Emergent themes were grouped together to form clusters which captured individuals’ accounts of experience and understanding. This was an iterative process for each transcript until clusters formed represented superordinate themes that could be applied across participants’ accounts, with emergent themes and initial clusters regularly revisited and modified or excluded as required. This process was done both within, and across individual transcripts until themes were reduced to those which most fully captured the quality of experience and understanding of participants ARMS. These themes were then discussed with members of the research team to check they reflected the overall content of participant’s accounts.

## Results

Data was analysed to address the two research questions. Three themes were identified to address the research question “How do individuals experience their ARMS?” and five themes were identified to address the research question “How do individuals understand the development of their ARMS?” These are presented separately below under the headings Experience themes and Understanding themes, and themes for each participant are presented in Table II.

**Table II.** Themes and participants.

		Participants				
	Super-ordinate Themes	Beth	Paul	Steven	Trevor	Joseph
Experience themes	Disturbed world/disturbed self	√	√	√	√	√
	Disconnection with the world	√	√	√	√	√
	Thunderstruck	√	√	√	√	√
Understanding themes	Absence of understanding	√	√		√	√
	Use of others	√	√	√	√	√
	Use of identity to form understanding	√	√	√	√	√
	Forming links	√	√	√	√	√
	Fragmented understanding: *Competing understandings*	√	√	√		√
	*Rejection/avoidance of understandings*	√	√	√	√	√

### Experience themes

Three themes were identified in relation to how individuals experience their ARMS which are outlined below.

#### Disturbed world/disturbed self

All participants described disturbances in both how they experience the world around them and their experience of themselves. This took multiple forms and included perceptual and sensory disturbances, and disturbances in affect, with disturbances in both the world and self presenting as inextricably linked.

*“Oh I started seeing and hearing stuff, having some unusual smells, like, it, I, probably stress related, I’ve been very easily stressed from whenever I was younger”* **(Trevor)**

Here Trevor describes disturbances in his experience of the world, noting sensory and perceptual abnormalities which he tells us are unusual, demonstrating insight into the disturbances he experiences. Whilst trying to understand the reason for such unusual experiences he lets us know he has also struggled with stress from a young age. By describing disturbances in his experience of the world along with being easily stressed, he lets us know disturbances in his experience of the world and the self go hand in hand and are closely linked together.

*“I had also been seeing like demons, demon kind of objects taking my family away and stuff like that after my granda passed away. Because me and my granda were very, very close and that’s whenever the depression got even worse.”* **(Joseph)**

Here Joseph describes a frightening image of seeing demon type objects taking his family away, but also his struggle with his depression. From his description he links the death of his grandfather to both his visual disturbances and his low mood. He lets us know by describing the loss of his grandfather, and his visual disturbances and low mood together, that the experience of his world and self changed following this loss. His ARMS and the visual disturbances he describes could be conceptualized as an extension to the disturbances in the world and self he experienced as a result of his bereavement, describing images and accompanying affect which can be interpreted as being closely related to loss.

#### Disconnection with the world

Participants all described a disconnection from the world around them. This disconnection took multiple forms across participants but was present in all their accounts.

*“I was just sitting thinking just em maybe in my room or something just sitting thinking for maybe an hour or two hours just em you know, you don’t you know you’re just so kind of distant from everything, you’re in your own wee world sort of thing.”* **(Steven)**

Here Steven describes the actual experiencing of symptoms was as if he was in his “own wee world”, distant from everything as if he was stuck in his experience unable to bring himself out of his room or his mind.

*“…my old GP I went to him and again I found it really hard to, you know, em express and then he would be like oh there’s, he said there’s nothing wrong just go for a walk (laughs) and I was like right ok, so I felt really stupid and just didn’t talk to anyone about it for ages and then.”* **(Beth)**

Beth’s extract demonstrates how disconnection from the world was not just observed in participants’ accounts in relation to the experience of symptoms, but also in relation to others. Beth lets us know here how difficult it is to put words to symptoms and how she was misunderstood by others, resulting in her feeling unable to talk to others further about her symptoms.

*“I couldn’t do basic things anymore like take the dog out or anything, because I was so paranoid thinking that people were coming after me, and I broke down in front of my mum one day and told her.”* **(Paul)**

Here Paul describes the impact of symptoms and how he became withdrawn from the world, unable to do basic things anymore viewing the world as dangerous. He tried to manage his fears and anxieties alone until it reached a point where it became too much and he broke down in front of his mum.

#### Thunderstruck

All participants described symptoms being persistent, transient and appearing to come from out of nowhere at times and that they were unsettling, frustrating, scary and confusing.

*“…we were driving through LOCAL TOWN and I thought there was someone sitting on a bench and there’s eh, I’m pretty sure there is someone sitting on a bench, and then I turned and looked again and nothing was there. And then I drove up the road another wee bit and there’s another bench and the exact same thing was sitting there, and it wasnae there anymore and then I says to my ma, I was just like for fuck sake and she goes what, and she says, I says I’m seeing these visions again and she says it’s just your mind playing tricks on you, but I see them nearly every day, I see shadows nearly every day whenever I’m just walking about”* **(Joseph)**

Here Joseph describes both the transient and persistent nature of his experiences. He shares how unsettling and frustrating the experience of symptoms is as he questions the reality of what he sees and seeks solace from his mother.

*“…but then sometimes it’s scary because you know it seemed to come out of nowhere you know, my own, maybe something that I had said into myself earlier and I was maybe doing something then that, I would hear that again while I was focusing on something so it would be like where did that come from.”* **(Steven)**

Here Steven explains how symptoms can appear to come out of nowhere even when engaged in other things, which is an unsettling, scary, and confusing experience.

### Understanding themes

Five themes were identified in relation to how individuals make sense of the development of their ARMS which are outlined below.

#### Absence of understanding

Four participants described an initial absence of understanding. Participants struggled to find links or reasons for their symptoms and tried to normalize them as a way to reassure themselves.

*“Em, I felt like I was able to tell people about it but I kept telling myself, you know that it would go away and that it was just in my mind”* (**Paul**)

Here Paul shows how he did not understand his symptoms, trying to normalize them by thinking they were just in his mind and something that would go away.

*“…there is nothing I could really link to you know feeling, you know worried about something or you know sad about something you just, you know it happened, but em, yeah there’s not really any, I think mainly why is the thought, why it did happen or you know what’s happening (laughs) to the world”* (**Beth**)

Beth shows us here how she was unable to link symptoms to anything and they appeared to just happen, without any explanation. Unable to form links Beth was left with an absence of understanding, questioning the world and her experiences.

#### Use of others

Participants all described using others as a source of information to help develop an understanding of their experiences through friends, family members and use of services.

*“Well I, like when people, like, I, I, if I asked someone if they smelled anything and they’d say no and like hearing things or whatever”* (**Trevor**)

In this extract Trevor describes initially using friends around him to develop an awareness that what he was experiencing was different to others. This was a starting point to beginning to develop an understanding of his experiences as different or unusual

*“Em, and the only way my mum can really, I’ve asked my mum about it and the only way my mum can explain it to me is the fact that our whole family is Christians then its Satan trying to get to us, em through his own wee way but he’s just trying to get to me at the minute”* (**Joseph**)

Joseph describes using his mums understanding to begin to develop an understanding of his own which mirrored hers.

*“…talking to MY THERAPIST, like we have kind of made more sense of it you know just, I think we were kind of touched on like relationships with people you know was a, you know, a, a lot to do with em, you know like feeling stressed and I kind of just, in and like eventually just switching off cause I didn’t really want to be in that situation anymore, so that really you know made sense em for that to be like a trigger em.”* (**Beth**)

This extract from Beth shows how she used understanding presented to her from services to help identify triggers and reasons for her symptoms. Although her use of language suggests some uncertainty in this understanding she is using it as something that makes sense to begin to build her own understanding on.

#### Identity

Throughout all participants’ accounts a strong sense of a struggle between their identity and their symptoms appeared for each of them. Participants’ sense making incorporated a range of different aspects of identity including personality, genetics, being a teenager, and symptoms as part of who they were/are.

*“I’ve kind of, you know I’ve always sort of, you know been like this, you know like eh, I’ve always had periods where you know I worried too much and all and eh, throughout childhood really.”* **(Steven)**

Here Steven explains that his symptoms are a continuation of his sense of self and describes how he has always “been like this” since childhood. He forms an understanding of his symptoms in the context of being a worrier merging his identity with his symptoms.

*“I think it could be a mixture of the genetics again and stuff like that or a chemical imbalance in my brain and stuff like that.”* **(Paul)**

Here Paul explains to us that his main way of understanding his symptoms is in relation to a biomedical model with genetic characteristic which are inherent in him. He draws on this understanding strongly throughout his interview telling the researcher his symptoms are part of who he is.

*“Yeah I always kind of put it down to just being like, I thought I’m just being dramatic or something or I’m being neurotic like or, em you know I was kind of like this is my fault you know, just get on with it kind of thing.”* **(Beth)**

Here Beth describes how initially she understood her symptoms were part of her personality as a teenager and describes how this then translated into self-blame and it being something that she did. This fusing of identity and symptoms appears across participants but as Beth’s understanding of her symptoms developed, she was more able to differentiate between her sense of identity and her symptoms.

#### Forming links

Participants described beginning to form understandings of symptoms by forming links both between symptoms and with life experiences. Forming links appears to be a tentative process where participants take a step back from their experiences and begin giving more considered thought to their occurrence and onset.

*“I would hear it more when I’m depressed than I would when I’m not depressed, I would still hear it when I’m not depressed but.”* (**Paul**)

Here Paul shows how he has begun to form links between his mood and his auditory disturbances. Although he has begun to form links between symptoms this is tentative in nature as auditory disturbances still occur irrespective mood.

*“I think that it’s just something that I would hold separate. Em, my dad leaving and my granda dying, it does trigger a thought of why I’ve got the depression so it does, it does trigger why I’ve got the depression. The anger issues stems from my da and what has happened to my mum. So there’s two things that add together for my anger issues. So, I do try and think what the fuck em let me see that adds up to that, that adds up to that.”* **(Joseph)**

Here Joseph echoes the tentative nature of forming links by considering chosen life experiences and holding others separate in relation to his mood.

#### Fragmented understanding

Throughout all participants accounts a fragmented understanding appears which developed from initial absence of understanding. This takes two forms.

##### Competing understandings

Given the initial absence of, and subsequent multiple ways and sources used to form understandings, participants struggled to merge these together and moved backwards and forwards between different understandings resulting in what appears to be incomplete or fragmented understandings.

*P:* “Em, well, eh, I don’t think the fire would have been that much of, it was just, yeah eh, it was nearly just the job I would say was the start of it. You know just, I don’t know I just kind of stayed quite during the whole fire thing….”

*I:* “Maybe I picked you up wrong, did you say that you said to the STEP team that you think that that has had some sort of impact or some sort….”

*P:* *“Yeah, em, it’s hard, it’s hard to know I mean, you know seeing the fire when I was younger and then seeing it like recently you know em, you know it brings, it did bring a link back you know.”* **(Steven)**

Here Steven illustrates competing understandings well demonstrating the difficulty merging his main understanding of stress caused by starting work with the impact of fires he has witnessed which he has gained from engaging with services. Although acknowledging both as contributing factors he is unsure of how to fit these competing understandings together.

##### Rejection/avoidance of understandings

During the interviews participants were given an opportunity to discuss and explore a range of different ways to understand the development of their ARMS. However all participants rejected different understandings throughout interviews by directly refuting them or using defences to cease further exploration.

*I:* “Okay. I’m wondering as well because you are saying there’s that element of understanding within being Christian, I mean is your sister…”

*P:* “My whole family is Christian”

*I:* “So I’m wondering do you ever think why you have them and maybe your sister doesn’t have them or maybe why other people might not have them, or is that something that you think about?”

*P:* “Not really. It’s never, it’s never something that I’ve thought about, but I’ll probably end up going home and start thinking about it (laughs). If I, if I think of anything else then sure I can always ring you and let you know”

*I:* “No I mean it’s…”

*P:* *“If you want to know anything else then you can always give me a ring and find out.”* **(Joseph)**

This extract from Joseph show the defences he employed when asked to further explore how his Christian faith was a reason for him to experience visual disturbances. He rejected further exploration of this both rejecting and avoiding the possibility of other understandings to emerge, although in some way he acknowledges his tentative understanding of symptoms didn’t fully fit the entirety of his experiences as it required further thought.

## Discussion

The importance of gaining a deeper understanding of the experiential dimensions of presenting difficulties has been highlighted as a key step for professionals to gain a deeper understanding of the phenomena they are dealing with (Nelson et al., 2008). This study set out to examine the subjective experience and understanding of the development of the ARMS. The analysis produced eight themes in total, three themes of experience (disturbed world/disturbed self, disconnection with the world, and thunderstruck) and five themes of understanding (absence of understanding, use of others, identity, forming links, fragmented understanding) which are discussed below. Similarly to Hirschfield et al.’s (2005) findings in their FEP study, themes were interwoven and participants described attenuated psychotic symptoms and their occurrence, mood difficulties, altered interactions with the world, and the emotional impact of symptoms. Understandings were formed and shaped by participants experience of symptoms, their sense of identity in the context of these, people and systems around them, and the difficulty managing multiple and competing understandings.

Disturbed world/disturbed self related to disturbances in how participants experienced the world around them, with all participants describing disturbances in the world and self as co-occurring and inextricably linked. Although UHR criteria do not directly identify mood difficulties, this appears to be an important aspect of the ARMS. Previous research in psychosis has identified the pain caused by symptoms and the losses associated with them (McCarthy-Jones et al., 2013), but participants accounts in this research highlight difficulties with mood are not just as a consequence of symptoms, but perhaps an integral part of presenting difficulties which require substantial clinical attention. This is in line with Garety et al.’s (2001) cognitive model of the positive symptoms of psychosis, which highlights the central role of emotion in the development of psychotic appraisals. However given the results from Fusar-Poli et al.’s (2014) recent review, which found comorbid affective disorders were not predictive of later transition to psychosis, this is an area which requires further consideration and research to allow for the development of theory for this specific clinical population.

The theme thunderstruck described the occurrence of symptoms and the emotional impact of these. The emotional impact of symptoms is not surprising given the disturbances in how participants experience the world around them. Previous research has focused on the pain of losses associated with psychosis (McCarthy-Jones et al., 2013) and the traumagenic distress of FEP and individuals experiences of treatment (Dunkley, Bates, & Findlay, 2015). However no previous research has subjectively reported how the experience of the ARMS can be disconcerting. Acknowledging the disconcerting nature of symptoms, and how participants described the occurrence of these, gives important insight into the subjective experiential dimension of the ARMS, allowing professionals working with this client group to gain a deeper awareness, and understanding, of what symptoms are like.

The theme disconnection with the world describes both the experience of symptoms, but also the difficulty participants described in connecting to others, resulting in a tendency to keep the experience of their ARMS to themselves. Previous research in the ARMS has reported participants describing isolation, difficulty with social interaction, and a tendency to keep experience of symptoms to themselves (Byrne & Morrison, 2010; Welsh & Tiffin, 2011), and retrospective accounts of the psychotic prodrome are characterized by social withdrawal, a loss of self which often results in job loss or academic failure (Boydell et al., 2010), and losses in self, consensual reality, security and relationships (McCarthy-Jones et al., 2013). This research mirrors previous findings in ARMS and psychosis studies, but highlights the difficulties of being unable to put words to symptoms and being misunderstood as a consequence. Being unable to find the words to explain symptoms undoubtedly makes it more difficult to approach others for help, especially when having previous experiences of feeling misunderstood.

With regards to how participants understood the development of their ARMS, five themes were identified which were interwoven and reflected an on-going process of forming an understanding, with each participant on different points in their journey with regards to this. This process is similar to what Williams and Healy (2001) described as forming exploratory maps of understanding mental illness, where participants drew on a range of co-occurring and at times contradicting understandings. Themes of understanding also reflect the on-going process of making sense of symptoms which is placed in the context of services, friends, and family.

The theme absence of understanding describes the initial stage of meaning making for participants, where each participant described being unsure of triggers or links between symptoms, and attempted to normalize these as a consequence. Judge and colleagues (2008) reported a similar process with FEP patients, where individuals notice a range of unusual experiences but do not necessarily translate these into a need for intervention. Furthermore, linking to the theme identity, participants described thinking symptoms were just part of who they were and tried to understand them in the context of the self, drawing on aspects of personality and viewing symptoms as just part of who they were. Linking the themes of Identity and Absence of Understanding together demonstrates the emerging fusion of symptoms with the self, as participants move from being unable to form an understanding, to understanding symptoms as part of who they are. This is similar to what Hirschfield and colleagues (2005) reported when they observed individuals’ explanations of their psychosis were often linked up to their sense of themselves prior to the onset of illness. The period of emerging adulthood which UHR criteria covers represents an important time for identity formation and development (Erikson, 1968). Participants understanding their symptoms as part of their identity may reflect this, as they begin to form more concrete ideas about their self, and move to developing greater autonomy from parents. However, as Harrop and Trower (2001) propose, the emergence of psychosis in adolescence may reflect blocks in adolescent development, as individuals struggle to form an identity separate from their family. Participants drawing on their identity as a way in which to understand the development of their symptoms may reflect this block in adolescence, as they struggle with their emerging identity, viewing symptoms as a continuation of, or fusing symptoms with, their sense of self.

The theme use of others highlights the importance of others, and that the on-going process of individuals making sense of their ARMS is placed in the context of services, friends, and family which surround them, who help form and shape emerging understandings. Participants all described using others as a way to begin to form an understanding of their symptoms, whether it be through first checking the reality of their experiences with friends or peers, to using services and family members to form links and develop narratives about the development of their symptoms. In many instances participants understanding of their ARMS also appeared to mirror that of family members, whether that be by considering the development of their ARMS due to internal biological factors, or by considering external factors and life events. This finding may relate to what Harrop and Trower (2001) propose, that psychosis which emerges in late adolescence represents a difficulty developing autonomy and individuation from family parents. Mirroring parent’s understandings of the development of their ARMS may represent the difficulty participants have in forming a separate mind from that of their parents, and the difficulties participants experience in developing autonomy and individuation.

The theme Forming Links provides further insights into this, where participants drew on a range of factors, including the use of others, to begin to develop narratives and understandings about the development of their symptoms. Participants in this study drew on a range of factors including relationship difficulties, experiences of trauma, personal characteristics, and difficulties managing the stresses of adolescence, such as school and adjusting to age relevant transitions like commencing employment. These are similar to findings reported in previous studies in psychosis (Dudley et al., 2009; Hirschfeld et al., 2005). Furthermore, participants began to form links not just with external environmental and relationship factors, but all also by beginning to from links with and between symptoms. These were tentative in nature and often related to mood. This is in keeping with the central role of emotion Garety and colleagues (2001) proposed in their theory of the positive symptoms of psychosis, where difficulties in affect were suggested to lead to psychotic appraisals of life events and unusual experiences. In keeping with this, participants in this study began to examine how their mood impacted on other symptoms they were experiencing such as paranoia or unusual visual and auditory disturbances. As such mood may represent the easiest place for individuals to begin to make sense of their ARMS.

The theme fragmented understanding combines the two minor themes: competing understandings and rejection/avoidance of understandings. Participants’ accounts in this study often represented incomplete or fragmented understanding as they struggled to merge a range of different sources of understanding together to form a coherent narrative to explain their symptoms. This is similar to Williams and Healy’s (2001) concept of exploratory models of understanding, with participants juggling multiple understandings (and absence of) from a variety of sources to begin to form narratives about the development of their ARMS. However these narratives often represented co-occurring and at times conflicting understandings. This appeared to lead to a friction between competing understandings which resulted in participants often rejecting or avoiding the emergence of understandings which were contrary to others. This is in contrast to the initial theme of absence of understanding, where participants had no explanation or personal understanding for the development of their ARMS, and further highlights a sense making process rather than a definitive way to understand of their experience of the ARMS. This is similar to what has been documented in previous studies of psychosis reviewed by Boydell and colleagues (2010), where young people engage in a search for meaning for their symptoms, adopting multiple explanations which change over time.

### Clinical implications

An important aspect of this study’s findings is that it gives insights into how individuals begin to form an understanding of the development of their ARMS. Findings highlight a sense making process which involves multiple, and at times conflicting understandings, gained from a variety of sources, including friends, family members, and services. As a consequence individuals manage a wide range of competing sources of information and understandings, resulting in the development of incomplete or fragmented understandings of their ARMS. An important aspect of clinical work may be to help individuals merge competing understandings together with multiple explanations initially needing to be considered by professionals, allowing clients to be understood from within their own systems of meaning. This also highlights the importance of working systemically with clients, and those around them, to merge a wide range of competing understandings together, and help form a more complete understanding of their ARMS. Developing a shared understanding between client, significant others, and services is likely to improve engagement and satisfaction with services, and strengthen therapeutic relationships (McCabe & Priebe, 2004).

Another important aspect of this study’s findings is that it adds to the current literature on the subjective experience of the ARMS. Findings highlight the emotional impact of symptoms for individuals, drawing attention to how disconcerting symptoms can be. This provides clinicians a deeper insight into the subjective experience of the ARMS and allows for a deeper understanding into the phenomena they are dealing with. This study also highlights mood as an integral part of presenting difficulties, which requires substantial clinical attention at both assessment and intervention stages, and further research is required to examine the role of mood in the ARMS more fully. This is important because mood is central to participants’ accounts of both experience and understanding of their ARMS, with participants in this study all describing experiencing difficulties with mood. Furthermore each participant used mood as a starting point to make links between life experiences and symptoms, and to make sense of other symptoms. From a phenomenological point of view, mood is not just a psychological, or mental state, but relates to disturbances in the body, and the intersubjective space in which we live, behave and act (Fuchs, 2014). For example, depression undermines individuals’ existential feelings of being with others, and leads to a sense of detachment and separation. These are often accompanied by cognitive symptoms; negative thoughts about the world, self and others. Given the overlap between the experience of mood and the ARMS, and in line with Garety and colleagues (2001) theory of the positive symptoms of psychosis, it is perhaps understandable that individuals sense making process involves reflecting on the impact of mood on their ARMS, with participants linking together disturbances in their emotional and mental states, intersubjective space and bodily sensations. As such mood may represent the easiest place for individuals to begin to make sense of their ARMS, and further highlights the importance clinically for attention to be paid to mood. One important aspect of clinical work may be to assist individuals in identifying the overlapping nature of mood and their ARMS, and how each impact upon the other.

Results from this study also highlight participants merging of their identity with their symptoms, suggesting that individuals’ understanding of their ARMS often gets interwoven with their own sense of identity. This adds to the findings of Hirschfield et al. (2005), and Judge et al. (2008), by suggesting symptoms are initially viewed as a continuation of individuals’ sense of self. Harrop and Trower (2003) argue that the development of psychosis involves a problem in the construction of self. The two major threats to this, which occur from the early formative years, are the unavailability of a mirroring other or the intrusive possession of the other. Such experiences may act to block the development of the subjective self, with individuals unable draw on significant others to provide the important feedback to enable the secure development of one’s own identity. Participants merging of their sense of self with their symptoms may reflect such difficulties, with an identity formed from early experiences which have not validated and scaffolded their own autonomous sense of being. This may have translated into participants difficulties separating symptoms from self, being left questioning “who am I?” and “what is me?” without autonomy or appropriate support from others. Helping assist individuals develop a coherent narrative, not only on the development of their symptoms, but also on their identity, may be of benefit given the tendency to view symptoms in the context of, or as a continuation of their sense of self. This, alongside the difficulty participants described in putting words to their symptoms, highlights the importance of education and early identification through public health campaigns to enable young people to both recognize, and seek help for their symptoms. Targeted mental health education programmes to help young people develop both an awareness of, and language and confidence to describe emerging mental health difficulties may enable them to both recognize the presence of, and seek help for their ARMS at an earlier stage. Assisting young people in developing the language to explain what seems unexplainable may be an important consideration for mental health education programmes in schools and systems which surround young people. Equipping young people with relevant ways to explain symptoms may assist them coming forward and feeling understood at an earlier stage, reducing the impact of symptoms on functioning, and reducing the length of time without appropriate support. Previous community based education and intervention programmes have demonstrated this as an efficient public health strategy which led to earlier referrals and preventative interventions (McFarlane et al., 2010). Educating not only young people, but also the professionals who work closest with them, such as teachers, GPs, and community workers, may also lead to quicker detection of symptoms and access to services. Recent research has demonstrated this as an effective method, with the Early Detection, Intervention and Prevention of Psychosis Programme (EDIPPP) adopting such an approach (Lynch et al., 2016). Lynch and colleagues model created a network of professionals, and community workers and members who were trained to identify the early signs of psychosis to generate rapid referrals to EDIPPP. Results demonstrated that outreach activities were correlated with increased referral rates across the six sites included in the study, supporting the feasibility, generalizability, and effectiveness of such an approach.

Finally, this study highlights the need for a unifying theory of the ARMS which would help both clinicians and their clients to collaboratively form a coherent understanding of their ARMS, and would guide future research in the area. Although findings from this study fit with both the cognitive theory of the positive symptoms of psychosis (Garety et al., 2001), which highlighted the central role of emotion in the development of symptoms, and the theory of blocked adolescence (Harrop & Trower, 2001), which considers psychosis as a consequence of difficulties establishing autonomy and individual identity, this study highlights that these theories provide a fragmented understanding which does not fully account for the subjective experience and understanding of the ARMS. This study points to the need for a unifying theory of the ARMS which accounts for factors associated with transition, and holds central the role of mood, identity, and the impact of systems around the individual, whilst keeping the subjective experience at the centre of its understanding. Factors which have been identified which increase the risk of transition include experiences of trauma (Bechdolf et al., 2010), lower levels of premorbid functioning (Yung et al., 2006), and low IQ (Ziermans et al., 2014). In contrast to this study’s findings, which highlights the importance of mood in the ARMS, although depression and anxiety are common comorbid presentations, and were previously indicated as predictors of transition (Johnstone et al., 2005; Yung et al., 2003), a recent meta-analysis reported they have no impact on transition (Fusar-Poli et al., 2014). To bring these findings together a unifying theory of the ARMS and transition to FEP is needed, which can help professionals and clients navigate forming an understanding of the ARMS, and the potential factors which may contribute to the development of FEP. This would also guide future research in forming further understanding into the factors and processes involved in the ARMS. To assist in theory development, further research in this area may wish to build upon some of the findings in this study by examining the role of identity formation in the ARMS, and further examine the importance of mood. Furthermore, prospective design studies may provide valuable insights into how individuals begin to merge competing and fragmented understandings together, and whether this is related to long term outcome, such as transition to psychosis or other measures of psychological well-being. It would be important to study people’s experiences over time of transition to psychosis, of improvement in ARMS and indeed the experiences of those whose ARMS remains stable over time.

### Strengths and limitations

Given the lack of theory about the development of the ARMS, this study highlights the ways in which individuals can begin to make sense of their symptoms. It draws attention to the important role of mood in both the presentation and understanding of the ARMS. The main strength of this study was that by utilizing qualitative phenomenological methodologies it ensured participants personal accounts were at the centre of this study’s findings, which were rich in subjective accounts of experience and understanding. The main limitation of this study was that information was not gathered on the therapeutic input participants had received prior to participating. Participants’ experiences of service involvement may have impacted on both their understanding and experience of their ARMS in comparison to those who have not received any therapeutic input. Comparing the subjective experience and understanding of the ARMS between individuals who have and have not had experiences of services may shed further on light on both the experience and understanding of the ARMS, and may be a useful direction for future research. Finally, this study’s exclusion criteria of non-English language participants means that the experience of individuals with an ARMS with differing language, cultures, and societal norms was not included. Future research including a wider range of participants may shed insight into the development of the ARMS within more culturally diverse populations and assist in developing a unifying theory of the ARMS.

## Conclusion

In conclusion, this study has added to the emerging literature on the subjective experience and understanding of the ARMS. It has highlighted the difficulties associated with both the experience and understanding of the ARMS, with competing and fragmented understandings developing in the context of identity, individuals and systems around the person, and mood and life experiences. Results draw attention to the lack of a unifying theory which explains the development of the ARMS. Clinically findings from this study highlight a number things: the importance of assessing and treating mood difficulties as part of the ARMS presentation; the need for targeted mental health education programmes in schools and systems which surround young people to assist in developing awareness and early identification of the ARMS; the role of services in assisting individuals merge competing understandings and form a coherent narrative, separate from their sense of identity, to help understand the development of their symptoms. Future prospective design studies may provide further insights into how individuals begin to merge competing understandings together and what relation this has to long term outcome. Further research may also wish to further explore the role of mood in the ARMS, and how individuals use it to begin to make sense of their symptoms.
